# Simulation-based learning experiences during clinical nursing education: insights from a qualitative document study

**DOI:** 10.1186/s12912-026-04668-y

**Published:** 2026-04-21

**Authors:** Line V. Pedersen, Kjersti M. F. Johnsen, Jørghild K. Jensen, Hilde Solli, Camilla Olaussen, Grith Overgaard, Inger Åse Reierson

**Affiliations:** 1 Department of Health and Nursing Science, University of Agder, Jon Lilletuns vei 9, Grimstad, 4879 Norway; 2 Faculty of Health Sciences, VID Specialized University, Oslo, Norway; 3 Department of Nursing, Lovisenberg Diaconal University College, Lovisenberggata 15b, Oslo, 0456 Norway; 4 Department of Nursing and Health Sciences, Faculty of Health and Social Sciences, University of South-Eastern Norway, Post Box 4, Borre, 3199 Norway; 5 Research group Clinical Competence in Nursing Education, University of South-Eastern Norway, Post Box 4, Borre, 3199 Norway; 6 Research group Interdisciplinary Professional Health, University of Agder, Jon Lilletuns vei 9, Grimstad, 4879 Norway

**Keywords:** Bachelor nursing education, Clinical placement, Nursing education research, Simulation-based learning experience, Study plan, Subject plan

## Abstract

**Background:**

Simulation-based learning experience is increasingly recognised as a pedagogical strategy that enhances nursing education by bridging the theory–practice gap and exposing students to complex clinical scenarios. Considering growing demands for registered nurses and the limited availability of clinical placements, simulation-based learning experience offers a structured and experiential learning approach. Although its relevance to clinical practice is well established, the extent to which simulation-based learning experience is integrated into subject plans for clinical placement courses remains insufficiently explored. The aim of this study was to explore how simulation-based learning experience was described in subject plans for clinical placement courses in Norwegian bachelor nursing programmes.

**Methods:**

This qualitative study was part of a nationwide initiative under the Norwegian Network for Educational Research in Nursing. Subject plans for clinical placements from all 13 Norwegian bachelor nursing programmes were collected. An explorative and descriptive design was employed, and a document analysis using manifest content analysis was conducted.

**Results:**

The analysis resulted in one overarching category – *Diversity in the level of description* – supported by four subcategories: *Not mentioned*, *Mentioned*, *Described to some extent* and *Described in detail*. The results revealed substantial variation in how simulation-based learning experience was documented. The lack of standardisation suggests that simulation-based learning experience is not systematically embedded in clinical placement planning.

**Conclusion:**

Simulation-based learning experience has the potential to strengthen clinical education, particularly in contexts with limited placement capacity or where students may not encounter essential learning scenarios. Explicit inclusion of simulation in subject plans for clinical placements may foster institutional commitment and contribute to more equitable and high-quality clinical learning experiences. Further research should be considered to examine how this pedagogical technique could be meaningfully integrated into subject plans for clinical placements.

## Background

To address the increasing global demand for registered nurses (RNs) [1], nursing education institutions face growing pressure to provide sufficient and high-quality clinical placements [2–4]. Clinical placements and clinical experiences are fundamental elements of the nursing education worldwide, serving to bridge the gap between theoretical knowledge and clinical application, and also to provide students with essential exposure to real-life healthcare situations and opportunities to develop core competencies [5, 6]. Globally, nursing students are required to complete a minimum number of supervised clinical hours in various clinical placements to complement their theory-based learning. In Australia, this minimum is 800 h, whilst in the United States, the required clinical hours vary by state, with no national regulatory board setting a minimum [7, 8]. Within the European Union (EU), Directive 2005/36/EC and its amendment 2013/55/EU mandate a minimum of 2,300 h of supervised clinical practice, constituting half of the total 4,600 educational hours required for completing nursing education [9]. As a member of the European Economic Area (EEA), Norway adheres to these directives, allocating 90 European Credit Transfer and Accumulation System (ECTS) credits to clinical practice within its total of 180 ECTS for the bachelor nursing degree. National regulations in Norway, introduced in 2019 and implemented from 2022, define the graduate competencies and academic expectations that are included in nursing education [10]. Each nursing programme is governed by a programme specific study plan that outlines the overall structure, pedagogical approaches and intended learning outcomes [10, 11]. Based on the programme-level study plans, individual course subject plans are developed for each course, including clinical placements courses. These subject plans specify the course-specific learning outcomes, content, required learning activities and assessment methods. They function as formal governing documents that outline what students are expected to learn and achieve during each clinical placement.

Due to increased student enrolment, limited site availability, and concerns about supervision and consistency [4, 12–14], quality in clinical practice might be compromised and nursing educators have turned to simulation-based learning experience (SBLE) as a way to provide rich learning experiences [15, 16]. Pedagogically, SBLE places students in learning situations that require them to actively direct and manage their own learning, thereby promoting sustained critical thinking and reflective habits [17]. A significant advantage of SBLE activities compared to clinical settings is that they occur in an environment where no actual patients are present, and thus no patients are placed at risk [17]. Any mistakes or decisions that students may make in SBLE activities present learning opportunities, enabling students to attain competencies through analysis and reflection. The use of SBLE activities in nursing education aligns with the Bologna process, in which the European educational institutions are encouraged to utilise active learning methods [18]. Because SBLE activities are experiential in nature [15, 17], experiential learning theory is a key pedagogical approach that is used to explain how SBLE can support or enhance nursing students’ clinical learning [19]. Experiential learning theory underscores the importance of integrating information gained through a concrete experience with pre-existing knowledge to create new knowledge and perspectives [20]. The elements presented in SBLE literature and simulation guidelines, such as the importance of active student engagement in the learning process, opportunities to reflect on an experience, contingencies for discussing ideas and concepts, and the importance of interaction between the student and the learning environment, are elements that are associated with enhanced learning in experiential learning theory [17, 20, 21]. Extensive evidence agrees that SBLE effectively prepares nursing students for real clinical settings, and has positive effects on students’ self-confidence, satisfaction, knowledge, critical thinking, general competence and clinical skills [15, 22, 23]. However, the strict EU requirement [9] is increasingly seen as a constraint, limiting the flexibility of educational institutions to adapt to evolving pedagogical methods during clinical studies [2, 24, 25]. SBLE provides learning experiences that are central to the development of professional competence [26], as structured, immersive scenarios replicate clinical settings, thereby allowing students to practice skills, clinical reasoning and decision-making in a safe environment [27, 28]. Evidence supports SBLE’s effectiveness in enhancing student confidence, critical thinking, satisfaction and competence [29–31]. Although simulation cannot fully replace learning through direct patient interaction, it can provide exposure to diverse learning experiences that may not be encountered during clinical placements [24, 32]. Studies also indicate that SBLE can enable students to complete key learning activities more efficiently than in traditional clinical placements. Sullivan et al. [33] found that students carried out a greater proportion of complex clinical activities in substantially less time in simulation compared to clinical settings. Several countries – including USA, United Kingdom and Australia – now permit partial substitution of clinical hours with simulation [34–36].

SBLE modalities include psychomotoric skill training, high-fidelity simulation, virtual reality and standardised patients [37–39]. In situ simulation—conducted in the students’ actual clinical placement settings in Norway, rather than in campus-based simulation centres—is particularly relevant in this context because it forms part of the pedagogical approaches that may be integrated into clinical placement courses [16, 40]. International standards, such as those set by the International Nursing Association of Clinical Simulation in Learning (INACSL) Standards Committee, guide the structure of SBLE activities, emphasising three core components: pre-briefing/briefing, simulation scenarios and post-simulation debriefing [21].

Despite growing evidence supporting the importance of SBLE in developing professional competence among nursing students, the opportunity for formal substitution of traditional clinical hours remains limited in Europe due to the EU directives [9]. Given SBLE’s demonstrated value in fostering clinical reasoning, reflective practice and skill acquisition [26], it has become particularly relevant to examine how SBLE as a pedagogical learning approach is incorporated in the formal structure of clinical studies in nursing education to strengthen the quality in clinical placements. This study therefore explored how SBLE was described and integrated in subject plans for clinical placement courses in Norway’s bachelor nursing programmes, where simulation cannot replace clinical hours but may serve as an important pedagogical approach supporting students’ learning.

## Methods

### Aim

The aim of this study was to explore how SBLE is described in the subject plans for clinical practice in bachelor-level nursing programmes in Norway.

### Design

An explorative and descriptive qualitative design was employed [41]. This design is appropriate when knowledge about a specific phenomenon is limited [42]. A document analysis of subject plans from the 13 bachelor nursing programmes in Norway was conducted, focusing on the inclusion of SBLE.

### Setting

This study was part of a nationwide Norwegian study conducted under the auspices of the Norwegian Network for Educational Research in Nursing (NorNERN) in which each of the 13 Norwegian educational institutions has a formal representative. The overall aim of the nationwide study was to explore and analyse the content of core curriculum documents addressing the pedagogical and nursing scope in clinical placement studies.

### Data collection

In accordance with the educational stipulations in the Regulation on the National Guideline for Nursing Education, which was enacted in 2019 [10], the first cohort of bachelor nursing students graduated in 2022. Data from the current study were collected from all 13 of the bachelor nursing programmes conducted in 2022–2023 in Norway. These comprised formal documents, including study plans and associated subject plans for each clinical placement throughout the respective three years of education. This represents a total of 13 study plans, one for each programme. The study plans gave rise to 72 subject plans, which covered all clinical studies throughout the respective three-year periods, distributed as follows: 13 1st-year subject plans, 29 2nd-year subject plans and 30 3rd-year subject plans. These subject plans covered clinical placements in nursing homes, home-based health services, medical and surgical hospital units and mental health services. The clinical placements included both municipal and hospital healthcare environments. The educational programmes were located at universities or university colleges throughout Norway. The formal leaders of the individual educational institutions provided informed consent in accordance with the ethical approval for access to relevant institutional documents. The study plans and associated subject plans for clinical placements were collected by designated representatives at the respective institutions and compiled into a centralised, digitalised database provided by NorNERN. This enabled all project groups to retrieve data relevant to their specific research scope.

In the Norwegian context, universities often operate campus-based simulation centres [37] and separate subject plans describing simulation centre activities are common. However, since the aim of this study was to analyse how SBLE is represented specifically within subject plans for clinical placement courses, we excluded documents that described SBLE solely in connection with campus-based simulation centres. These activities are typically organised through dedicated simulation-centre subject plans and therefore fall outside the scope of the present analysis.

### Data analysis

A manifest content analysis, inspired by Graneheim and Lundman [43], was employed to analyse the subject plans for clinical placements in 13 bachelor nursing programmes in Norway. Content analysis is a systematic approach for interpreting textual data by organising and categorising explicit statements into meaningful units. In line with a manifest content analysis approach, the analysis focused on the visible, surface-level content of the documents—what the text actually stated [43]. This involved repeated readings to identify meaning-bearing units, which were subsequently condensed. An abstraction process followed, whereby these condensed units were organised into codes, subcategories and one main category that captured the explicit manner in which SBLE was described across institutions [43].

The preliminary analysis included obtaining a holistic overview of the material, which included the overall study plan for each three-year programme and the subject plans for their respective clinical placements. All seven members of the research team actively participated in this phase of the study. The researchers collaborated in pairs, with each pair assigned responsibility for the analysis of two to three educational study plans and their corresponding subject plans, focusing specifically on identifying content related to the concepts, simulation or practical skills, both of which are included in SBLE [16]. The holistic view revealed that the data regarding SBLE in clinical placements were to be found in the subject plans, due to their level of concretising the study plans.

**Fig. 1:**
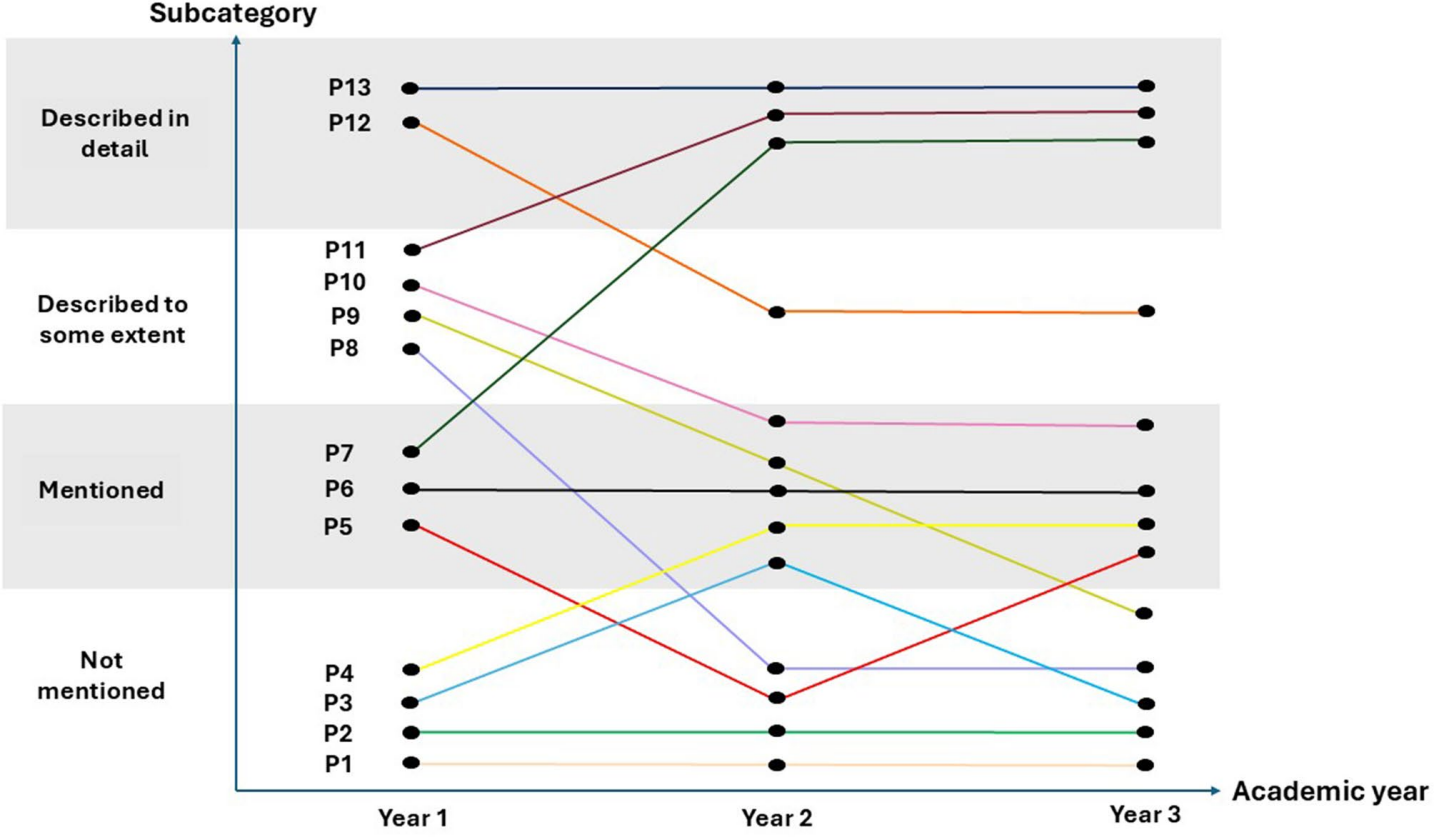
An overview of the descriptions of SBLE in subject plans for clinical placement studies for the 13 bachelor nursing programmes in Norway. Numbers with corresponding colours represent a separate part of the included bachelor nursing programmes. The letter P stands for ‘programme’. Subcategories (Y-axis) are outlined in relation to academic year (X-axis). In the following, the results will be presented in light of the four subcategories

To answer the research question, the research group (all seven authors) continued the analysis process by focusing solely on the subject plans for the clinical placements. After reading the documents, meaning units were identified and inductive codes were developed for each clinical placement subject plan. Researchers LVP, KFJ and IÅR developed preliminary subcategories, which were further discussed in the wider research group until agreement was reached. For further validation, the agreed subcategories were thoroughly assessed considering the data by LVP, KFJ and IÅR. For excerpts of the analysis process, see Table 1. During this phase, the research group identified the variations in how the subcategories emerged differently in each programme throughout the three years. Figure 1 visualises this in greater detail. In the final step, the research group discussed and agreed upon the main category. Virtual meetings were held to ensure necessary engagement during the entire process, and these were facilitated by Teams technology, which was also used to share documents. Microsoft Word and Excel were utilised in the analysis to create an appropriate overview.

**Table 1:** Excerpts from the analysis process

Codes	Sub-categories	Main category
Simulation not mentioned	Not mentioned	Diversity in the level of description
Simulation mentioned alone or as one of several learning activities	Mentioned	
Partial concretisation of the simulation content	Described to some extent	
Detailed descriptions of the type of practical skills	Described in detail	
Detailed description of the situation that can be simulated		
Detailed description of the skills/competences included in the simulation situation		
Detailed description of the content and structure of the simulation		
The simulation is explicitly coupled with the relevant subject matter learning outcomes		

Note: The codes that form each subcategory varied widely in scope—from brief references to SBLE to detailed descriptions of simulation content, competencies and structures. The subcategories represent the abstraction of this diverse set of manifest codes

### Rigour

To strengthen the trustworthiness of the analysis, we drew on the criteria of credibility, transferability, dependability and confirmability [44, 45]. To ensure credibility, researchers conducted repeated reflection during digital meetings throughout the analysis process. All researchers had several years’ expertise applying SBLE in nursing education, and five of the authors (KFJ, JKJ, HS, CO, IÅR) also had broad experience of qualitative research. Given that SBLE is an important area in nursing education and is intended to enhance nursing competence, the findings may have transferability to nursing education at an international level. Dependability was addressed by collecting all clinical placement subject plans for each of the 13 bachelor nurse education programmes in Norway, and by providing a transparent description of the research processes. Confirmability was based on data from the subject plans and visualised by providing extracts from these. The Standards for Reporting Qualitative Research (SRQR) guidelines [46] and the corresponding SRQR checklist [47] was applied to enhance rigour.

## Results

The result of the current study identified one main category and four subcategories that described how SBLE was portrayed in the subject plans for clinical placements in the Norwegian bachelor nursing programmes. The main category *Diversity in the level of description* encapsulated the variety in how the individual nursing education subject plans for clinical placements described SBLE. Although it is less common for a manifest content analysis to result in a single main category, this appeared analytically appropriate in our study, as the four subcategories reflected the manifest differences in the degree of detail rather than distinct conceptual domains [43]. This variation was reflected in the four subcategories: *Not mentioned*, *Mentioned*, *Described to some extent* and *Described in detail.* Such variation appeared not only across the nursing programmes, but also, in most cases, within the programmes’ individual subject plans for clinical placements. Figure 1 visualises this feature. Some institutions emphasised SBLE consistently throughout all three years, and some programmes fluctuated in their focus on SBLE, either from a lower to a more detailed level of description or vice versa. Two institutions omitted SBLE entirely in all three years in all subject plans for clinical placements.

### Not mentioned

This subcategory identified the subject plans in which SBLE was not mentioned. As illustrated in Fig. 1, the results showed a variation regarding the years in which SBLE was omitted (programme numbers P1, P2, P3, P4, P5, P8). In two programmes (P1 and P2), SBLE was absent in all academic years. In four programmes the subcategory was identified either once or twice across the three years of study. For instance, one programme (P4) did not mention SBLE in the first year; in another programme (P3), the subcategory was identified in the first and third years; one programme (P5), did not mention SBLE in the second year only; and finally in one programme (P8), the subcategory was identified in the second and third years.

### Mentioned

This subcategory highlights subject plans that mentioned SBLE either as a standalone concept or as one of various learning activities, without further elaboration. One example from a first-year subject plan reads: “*Lectures*,* seminars*,* literature studies*,* written assignments*,* reflection*,* case studies*,* [practical] skills training and practice*,* simulations and self-study*,* as well as the use of ICT [Information and Communication Technology]*” (P6). Although identified in seven programmes (P3, P4, P5, P6, P7, P9, P10), as shown in Fig. 1, there was considerable variation between these. One programme (P6) consistently used this level of description throughout all three years, whilst two others (P3, P5) presented this subcategory as the most detailed description of SBLE in their clinical placement subject plans (Fig. 1.). Two programmes (P3, P9) *mentioned* SBLE in the second year only, and two programmes (P4, P10) *mentioned* it in the second and third year. An example from a third-year plan illustrates this: “*The course may include self-study*,* lectures*,* group work with or without supervision*,* simulations*,* case-based learning*,* e-learning resources or seminars*” (P4). No further descriptions were provided.

### Described to some extent

In this subcategory, the programmes offered some specifications regarding SBLE, including descriptions of the simulation focus. An example from one programme stated: *“[…] the first week is preparation for practice with simulation as the main learning activity*,* related to the current clinical practice focus*” (P12). Another example reads: “*Practical skills in basic nursing and CPR*,* as well as focus on communication and interaction skills*” (P6). Another programme described SBLE *to some extent* only in the first year: “*Mandatory participation in teaching related to practice preparation*,* skills training and simulation*” (P10). As seen in Fig. 1, this subcategory was primarily used in the first year of education. Four programmes (P8, P9, P10, P11) employed this level of description in the first year, and one programme employed this level of description in the second and third year (P12).

### Described in detail

Programmes within this subcategory provided comprehensive descriptions of SBLE in their subject plans for clinical studies. These included specification of the practical skills that were to be acquired through simulation, the design and content of simulated scenarios, and the alignment of simulation activities with the intended learning outcomes. The description of the learning arena in which SBLE took place was also included in two of the programmes. One (P13) maintained this level of detail throughout all three years in their subject plans for clinical placements. Another (P12) provided a *detailed description* of SBLE in the first year only: “*The simulations include a briefing phase*,* scenario execution and a debriefing phase. The debriefing consists of a description phase*,* an analysis phase and an application phase. The content of the scenarios includes training on basic skills related to learning outcomes*,* as well as a description of the scenario and learning outcomes”* (P12). Two programmes (P7, P11) offered detailed descriptions for the second and third years only. For example: “*Head-to-toe [physical] examination on one another and performing an aseptic procedure. Simulation: ABCDE [Airway*,* Breathing*,* Circulation*,* Disability and Environment] / ISBAR [Identification*,* Situation*,* Background*,* Assessment*,* Result] / acute situation. Individual written reflection after simulation*” (P11).

In addition to specifying the content and simulation modalities, two of the subject plans (P12, P13) described clinical education as comprising both SBLE conducted at simulation centres and the experience gained in clinical placement settings. The plans specified in detail the distribution of time between these two arenas, detailing the number of hours or weeks allocated to simulation activities in the simulation centres (as pre-clinical or post-clinical studies) and to clinical placements. Such clear descriptions as to where SBLE took place are illustrated by the following example: “*Skills training is conducted in the simulation centre. The training includes four thematic stations: blood transfusion*,* enteral feeding tube placement*,* stoma care and surgical wound management. During the clinical placement period*,* students participate in three practice groups. One of these takes place in the simulation centre and involves simulation-based patient scenarios aligned with the intended learning outcomes of the course”* (P13).The level of specificity is further exemplified: “*Skills training is conducted in the simulation centre*,* including one day dedicated to injections (subcutaneous and intramuscular) and one day focused on catheterisation (urinary catheter insertion)*” (P13). For one education programme, concrete SBLE was described during clinical studies. An example from the third year shows: “*Simulation in palliative care within home-based services*,* lasting 2 days*” (P7). Nevertheless, it was not further described whether this SBLE took place as an in situ simulation or at a simulation centre.

Learning outcomes were also explicitly addressed for the SBLE. In the pre-clinical studies that were included in the subject plans for clinical placements, guiding a fellow student in three different procedures was one learning outcome: “*Supervision of a fellow student in three different procedures: subcutaneous injections*,* intramuscular injections and permanent catheterisation. The theme is clinical leadership in teams with scenario-based simulation*” (P13). This example demonstrates how SBLE not only supported the development of practical skills but also provided a learning arena for peer-to-peer interaction and the advancement of leadership competencies, thereby addressing more complex learning outcomes.

To provide additional detail, the subject plans referred to supplementary information available on the programme’s digital learning platform: “*Information regarding the thematic content and implementation is provided via Canvas [digital learning platform]*” (P13).

## Discussion

This study explored SBLE representation in subject plans for clinical placements in Norwegian nursing education programmes. The results revealed considerable variation in how SBLE was described, with a general tendency towards limited documentation. Although some institutions provided comprehensive descriptions of simulation activities aligned with intended learning outcomes, others only briefly mentioned SBLE or omitted it entirely. This inconsistency indicates that, although SBLE may serve as a valuable learning activity during clinical placements, it is not positioned as a systematic component of the clinical placement curricula. This warrants attention given the extensive body of research demonstrating the pedagogical value of simulation for enhancing clinical competence in nursing education [28, 31, 48].

To interpret these findings, the results are discussed in the light of three broader challenges in clinical education, as outlined in the background: the positioning of SBLE as a strategic response to challenges in clinical practice; the role of in situ simulation and the imperative for curriculum transparency; and the broader policy landscape, including institutional autonomy and its implications for curricular coherence.

### SBLE as a strategic response to practice challenges

The marked variability in how SBLE was described in the subject plans suggests that SBLE is not consistently positioned as pedagogical strategy to address known challenges in clinical placements. Given concerns regarding uneven access to learning opportunities during clinical placements [4, 49], the absence of clear SBLE descriptions in many subject plans may indicate missed opportunities in providing support to students in developing essential competencies. Across institutions, SBLE was either omitted, mentioned only superficially or described without alignment to intended learning outcomes. This inconsistency warrants attention, as unclear or fragmented SBLE integration may contribute to inequitable learning experiences. When placement quality and exposure vary, students may not encounter clinical situations that are foundational for developing critical thinking, clinical reasoning, ethical judgement and psychomotor skills [40, 50, 51]. Without strategically integrated SBLE to compensate for these gaps, key learning opportunities risk becoming dependent on placement availability rather than curricular design. Purposefully designed SBLE might help mitigate this variability by enabling students to experience realistic, high-risk or infrequent scenarios in a controlled learning environment [52]. Applying such experiences in situ can help to ensure that, regardless of placement context, students have opportunities to develop essential competencies while conducting their clinical studies [40, 53]. Integrating high-quality simulation alongside traditional practice may therefore enhance the consistency and quality of students’ clinical learning and support their development of professional competence [5, 26, 32]. This becomes increasingly important in light of the growing demands placed on clinical sites and supervision resources [34, 36].

Although the EU directive [9] emphasises the importance of structured and comprehensive clinical education, they do not prescribe specific pedagogical approaches such as SBLE. The lack of explicit policy guidance endorsing SBLE as a valuable approach to experiential learning – despite evidence supporting its effectiveness [19, 20] – may partly account for its limited visibility within national regulatory requirements [10]. This, in turn, may contribute to the variation observed in how SBLE is implemented across clinical education settings, depending on institutional priorities, available resources and individual educators’ expertise. Although adaptation to local contexts is important [51], it may lead to divergent learning opportunities and challenge the overarching goal of nursing education to meet national requirements that are intended to ensure the consistent achievement of learning outcomes across educational sites [10]. In the absence of formal policy requirements or coordinated implementation plans, institutions may differ considerably in how and to what extent they incorporate SBLE into clinical education. Such variations may present challenges to ensuring equitable access to high-quality learning opportunities, both within and across national contexts.

### The role of in situ simulation and curriculum transparency

The vague, limited and inconsistent description of SBLE in most of subject plans in our study may lead to nursing students missing opportunities to participate in in situ simulations, alongside experienced healthcare professionals such as physicians and nurses [54]. When explicitly included in curriculum documents, learning activities become part of formal planning processes and are more likely to be implemented systematically [11]. In Norwegian nursing education programmes, both the national framework and institution-specific course plans are binding and serve to concretise regulatory requirements, thereby governing the structure and content of teaching [10]. However, the absence of explicit references to in situ SBLE may reflect uncertainty or caution regarding commitment to resource-intensive educational activities. Given the persistent challenges in securing sufficient and high-quality clinical placements, educational institutions and policymakers may need to consider a stronger commitment to integrating SBLE within planning and regulatory frameworks [55]. When simulation is clearly articulated in curriculum plans, it conveys institutional responsibility and pedagogical intent [51]. Such transparency may facilitate effective planning and resource allocation, and ultimately contribute to more consistent and equitable learning experiences for students [17]. The variations and limited emphasis on SBLE found in our study may contribute to uncertainty about whether newly graduated nurses consistently achieve the expected clinical competencies. This uncertainty could, in turn, be associated with the elevated stress levels reported among newly graduated nurses as they transition into professional practice [56, 57]. Among other factors, this stress has been linked to insufficient practical skills competencies in newly graduates [5]. Integrating in situ simulation into the subject plans for clinical studies may help address this gap by providing structured opportunities for experiential learning in realistic settings [20, 40].

Some of the subject plans (P13, P12, P11, P7) in our study incorporated SBLE in the subject plans for clinical studies; however, a distinct separation was described between the hours allocated to clinical placements and those allocated to SBLE in a simulation centre. This may reflect an intention to demonstrate alignment with the EU directive [9]. However, the absence of similar detail regarding the clinical component, particularly the use of in situ simulation, leaves uncertainty about whether and how such methods are implemented in practice.

### Policy context and institutional autonomy

The EU directive [9] impose a strict requirement for clinical placement hours in nursing education. Nordic educators have called for revisions to the directive to allow greater flexibility in the delivery of clinical education. They argue that the quality of clinical placements should inform both the content and the pedagogical approaches employed, including the expanded use of SBLE [2, 55]. The study by Hayden et al. [34] provided evidence to support that high-quality structured simulation could replace up to 50% of traditional clinical hours without compromising learning outcomes. Central stakeholders in Norwegian nursing education have participated in the discourse revolving around the premise that there is no concrete evidence proving that at least half of the hours in nursing education must be dedicated to clinical studies for students to successfully attain the necessary learning outcomes [58–60]. This raises important questions about whether a more flexible approach – incorporating alternative effective teaching methods – could yield equally robust educational outcomes.

### Limitations

A limitation of this study concerns the interpretation of curriculum documents at different levels. While the national regulation for nursing education in Norway [10] and the institutional subject plans are legally binding, we acknowledge that additional documents exists at levels below the subject plans, such as time schedules and internal guidelines. These documents may provide further insight into how national guidelines are operationalised in practice. Because these documents were not publicly available, our analysis may not fully capture the extent to which simulation-based learning experiences (SBLE) are systematically implemented during clinical placements. Consequently, the findings primarily reflect the formal intentions expressed in publicly accessible documents rather than the actual practices within institutions. The findings might have differed had it been possible to conduct first-hand interviews with campus or hospital leaders.

## Conclusion

The findings of this study revealed substantial variation in the extent and manner in which SBLE was described in subject plans for clinical placements. We suggest that a more explicit integration and systematic documentation of SBLE as a pedagogical approach within clinical placement curricula may contribute to improving the overall quality, coherence and equity of nursing education. SBLE could serve as a valuable supplement in contexts where access to clinical placements is limited or where students may not encounter essential learning scenarios during traditional practice. By incorporating structured simulation activities – particularly in situ simulations involving experienced clinicians – educational institutions might help students gain exposure to critical clinical situations, thereby potentially enhancing their preparedness and clinical competence. Furthermore, the explicit inclusion of SBLE in curriculum documents may encourage institutional commitment, support planning, and promote more equitable and high-quality clinical learning experiences.

## Data Availability

Data supporting the findings of this study are available upon request from the corresponding author.

## Abbreviations

ECTS: European Credit Transfer and Accumulation System
EEA: European Economic Area
EU: European Union
NorNERN: Norwegian Network for Educational Research in Nursing
R&D resources: Research and development resources
RN: Registered Nurse
SBLE: Simulation-based learning experience
